# Relaxometry with Nitrogen Vacancy (NV) Centers in Diamond

**DOI:** 10.1021/acs.accounts.2c00520

**Published:** 2022-12-07

**Authors:** Aldona Mzyk, Alina Sigaeva, Romana Schirhagl

**Affiliations:** † Groningen University, University Medical Center Groningen, Antonius Deusinglaan 1, 9713AW Groningen, the Netherlands; ‡ Institute of Metallurgy and Materials Science, Polish Academy of Sciences, ul. Reymonta 25, 30-059 Kraków, Poland

## Abstract

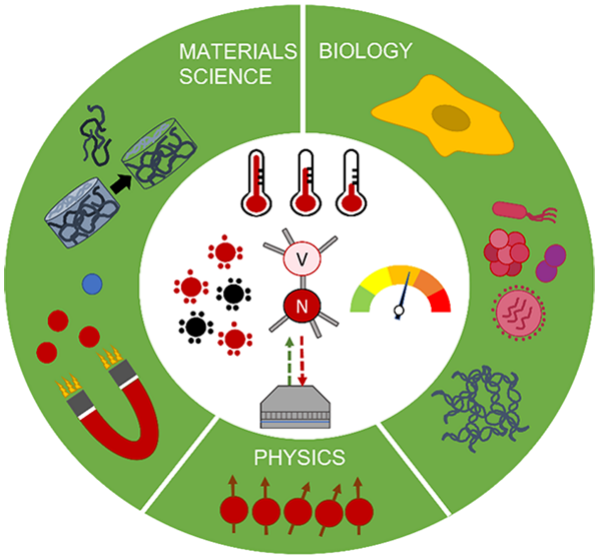

Relaxometry is a technique which makes use of a specific crystal lattice defect in diamond, the so-called NV center. This defect consists of a nitrogen atom, which replaces a carbon atom in the diamond lattice, and an adjacent vacancy. NV centers allow converting magnetic noise into optical signals, which dramatically increases the sensitivity of the readout, allowing for nanoscale resolution. Analogously to T1 measurements in conventional magnetic resonance imaging (MRI), relaxometry allows the detection of different concentrations of paramagnetic species. However, since relaxometry allows very local measurements, the detected signals are from nanoscale voxels around the NV centers. As a result, it is possible to achieve subcellular resolutions and organelle specific measurements.

A relaxometry experiment starts with polarizing the spins of NV centers in the diamond lattice, using a strong laser pulse. Afterward the laser is switched off and the NV centers are allowed to stochastically decay into the equilibrium mix of different magnetic states. The polarized configuration exhibits stronger fluorescence than the equilibrium state, allowing one to optically monitor this transition and determine its rate. This process happens faster at higher levels of magnetic noise. Alternatively, it is possible to conduct T1 relaxation measurements from the dark to the bright equilibrium by applying a microwave pulse which brings NV centers into the −1 state instead of the 0 state. One can record a spectrum of T1 at varying strengths of the applied magnetic field. This technique is called cross-relaxometry. Apart from detecting magnetic signals, responsive coatings can be applied which render T1 sensitive to other parameters as pH, temperature, or electric field. Depending on the application there are three different ways to conduct relaxometry experiments: relaxometry in moving or stationary nanodiamonds, scanning magnetometry, and relaxometry in a stationary bulk diamond with a stationary sample on top.

In this Account, we present examples for various relaxometry modes as well as their advantages and limitations. Due to the simplicity and low cost of the approach, relaxometry has been implemented in many different instruments and for a wide range of applications. Herein we review the progress that has been achieved in physics, chemistry, and biology. Many articles in this field have a proof-of-principle character, and the full potential of the technology still waits to be unfolded. With this Account, we would like to stimulate discourse on the future of relaxometry.

## Key References

- NieL.; NusantaraA. C.; DamleV. G.; SharminR.; EvansE. P. P.; HemelaarS. R.; Van der LaanK.; Perona MartinezF. P.; VedelaarT.; ChipauxM.; SchirhaglR.; LiR.Quantum monitoring of cellular metabolic activities in single mitochondria. Science Advances2021, 7, eabf05733413874610.1126/sciadv.abf0573PMC8133708.^1^*Here we demonstrate quantum sensing with relaxometry in single isolated mitochondria, as well as mitochondria in their cellular context*.
- NieL.; NusantaraA. C.; DamleV. G.; BaranovM. V.; ChipauxM.; Reyes-San-MartinC.; HamohT.; EpperlaC. P.; GuricovaM.; CiglerP.; Van den BogaartG.; SchirhaglR.Quantum sensing of free radicals in primary human dendritic cells. Nano Lett.2022, 22, 1818–18253492908010.1021/acs.nanolett.1c03021PMC8880378.^2^*This is the first demonstration of relaxometry in primary cells. More specifically, we investigated stress responses in dendritic cells*.
- Reyes-San-MartinC.; HamohT.; ZhangY.; BerendseL.; KlijnC.; LiR.; KawalkoJ.; MzykA.; SchirhaglR.Nanoscale MRI for Selective Labeling and Localized Free Radical Measurements in the Acrosomes of Single Sperm Cells. ACS Nano2022, 16, 10701–107103577198910.1021/acsnano.2c02511PMC9331174.^3^*Here we use relaxometry to differentiate radical formation at different subcellular locations in boar sperm during sperm maturation.*
- WuK.; VedelaarT. A.; DamleV. G.; MoritaA.; MougnaudJ.; San MartinC. R.; ZhangY.; Van der PolD. P. I.; Ende-MetselaarH.; Rodenhuis-ZybertI.; SchirhaglR.Applying NV center-based quantum sensing to study intracellular free radical response upon viral infections. Redox biology2022, 52, 1022793534992810.1016/j.redox.2022.102279PMC8965164.^4^*Here we use relaxometry to investigate the free radical generation a virus particle is experiencing during a host cell response to a viral infection.*

## Introduction

**Figure 1:**
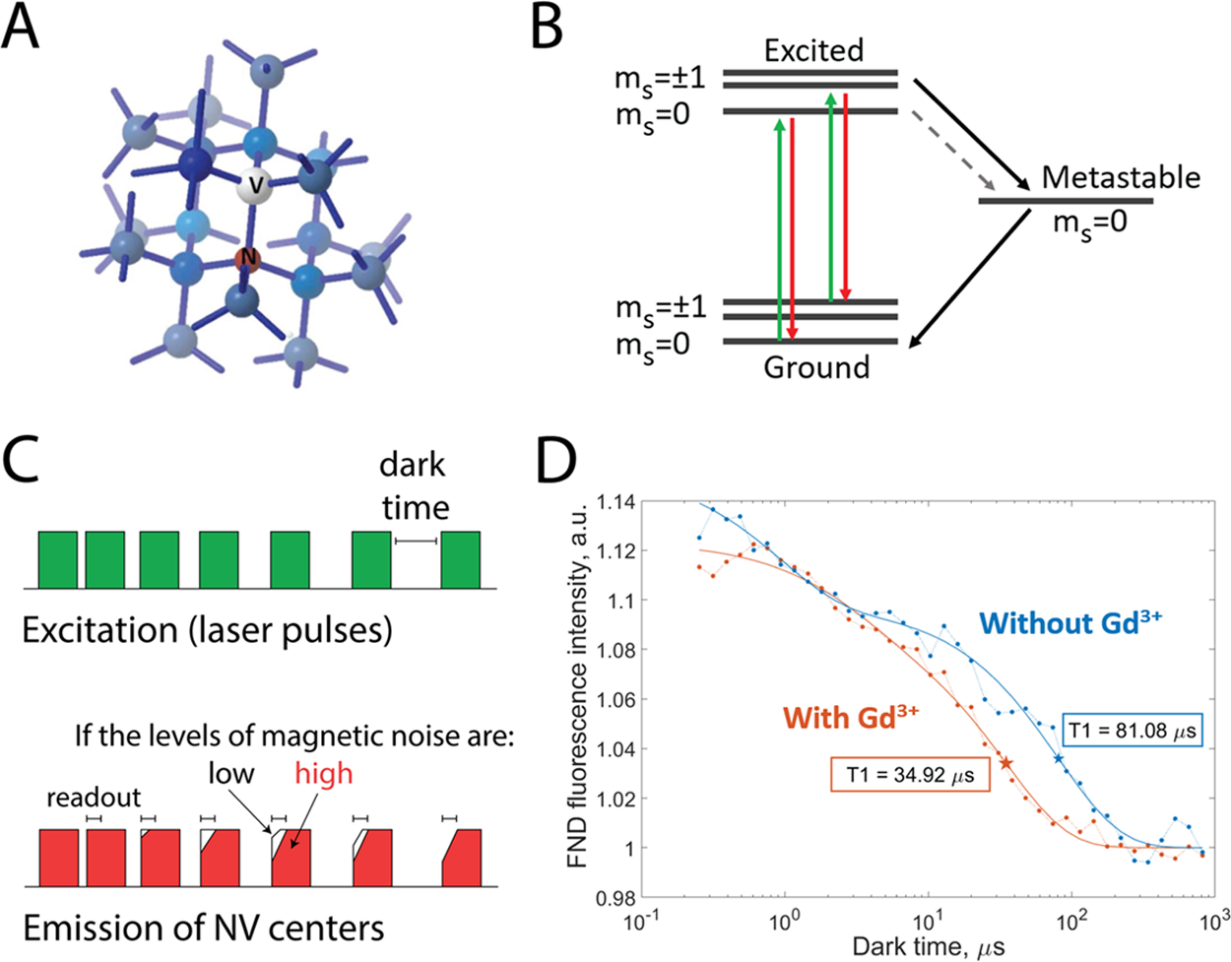
Foundation of NV center based relaxometry. (A) Nitrogen vacancy centers are defects of the diamond crystal structure: a nitrogen atom is present instead of one of the carbon atoms, and one of the neighboring positions in the lattice is vacant. (B) A diagram of the NV center’s energy levels. (C) The NV centers are pumped into a brighter ground state with a green laser pulse. The laser is switched off for a predefined period of time (dark time), and the NV centers are allowed to relax back to the darker equilibrium state. The observed fluorescence becomes less bright as the dark times increase. The transition from the bright polarized state to the darker equilibrium state happens faster at higher levels of magnetic noise (e.g., due to paramagnetic species). (D) The NV center’s fluorescence intensity plotted against the corresponding dark times can be used to calculate the T1 relaxation constant. If the relaxation is slower, one observes longer T1 values (blue; diamond without Gd^3+^). Faster relaxation can happen due to the changes in the NV center’s environment, such as the presence of paramagnetic species. In this case, the T1 curves are shifted to the left, producing shorter T1 values (orange; after adding paramagnetic Gd^3+^ ions).

Diamonds are widely used and known for their inertness, hardness, and high refractive index. In the last decades, color centers in diamonds have been increasingly popular due to their unique magneto-optical properties.^1−8^ Among a wide range of possible color centers described in diamond, the NV center (shown in Figure 1A) is the most prominent defect.^9^ NV centers exhibit fluorescence in red and far red regions upon excitation with a green laser. Since the defect is protected within the crystal lattice, it is prevented from bleaching and thus infinitely photostable, which is highly desired for applications in labeling. NV centers also have spin states that can be manipulated optically or with microwaves and can be differentiated by their fluorescence.^10^

This Account is about a specific way to use these properties for sensing, a technique called relaxometry. Relaxometry experiment starts with polarizing the spins of NV-centers in the diamond lattice into a bright ms = 0 ground state, using a strong laser pulse. Afterward the laser is switched off and the NV-centers are allowed to stochastically decay into the equilibrium mix of different magnetic states (ms = 0 and ms = +/–1). The polarized configuration exhibits stronger fluorescence than the equilibrium state, allowing one to optically monitor this transition and determine its rate. This process happens faster at higher levels of spin noise, and the relaxation rate is concentration-dependent. The pulsing sequence that is used for relaxometry measurements as well as a typical result are shown in Figure 1C,D. The spin relaxation dynamics follows a decreasing exponential whose time constant is called the spin relaxation time, or T1. However, due to the short-range of the spin interactions, T1 here is only sensitive to magnetic noise within nanoscale voxels around the defect. Alternatively to this most basic scheme, it is also possible to add a microwave pulse, which brings the NV centers into the ms = −1 or +1 state.^11^ As a result, one can observe relaxation from the dark to the brighter state. This scheme has the striking advantage that one can rule out artifacts that occur if the charge state of the NV center switches (a change from NV^–^ to NV^0^ might be interpreted as shortening of T1 since NV^0^ is less bright) or bleaching of background fluorescence. Performing T1 in the presence of microwaves, however, increases the complexity of the equipment and causes heating to the sample, which is often undesired.

Relaxometry was first demonstrated in 2013 when it was demonstrated that the relaxation time is reduced in the presence of paramagnetic ions.^12^ Since then, relaxometry has been used for many different applications in sensing, which will be reviewed in this Account.

## Cross-Relaxometry

Cross-relaxometry, a technique that has first been shown by Wood et al., allows one to reveal spectral information with relaxometry.^13^

**Figure 2:**
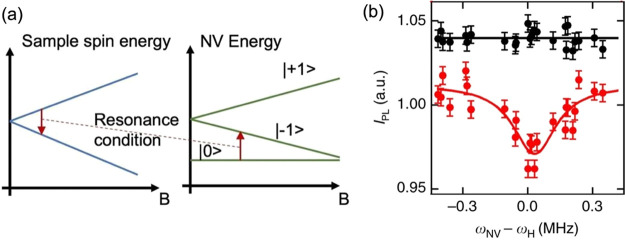
Cross-relaxometry principle. (a) Cross-relaxometry offers spectral information. If T1 experiments are done in the presence of a varying magnetic field **B**, there is a condition where the energy difference between the NV center energy levels equals the difference in the energy levels of the spins in the sample. When the magnetic field is swept, there is a peak at this energy which is characteristic for the sample spin. (b) Example spectrum taken with the cross-relaxometry method. The scan over the Larmor frequency of 1H is shown in red. Data from ref (13).

If a magnetic field is applied to NV centers the ms = +1 and ms = −1 are not equal in energy any more. The difference between these states increases with increasing magnetic field. By varying the magnetic field, one can also adjust the difference between the ms = 0 and the ms = −1 or +1 states. If this energy matches the transition energy of spins in the surrounding environment, the T1 is even further reduced. Thus, sweeping the magnetic field while recording T1 reveals spectroscopic information. The energy diagram that explains the cross-relaxometry principle is shown in Figure 2.

## Different Ways of Conducting Relaxometry Experiments

**Figure 3:**
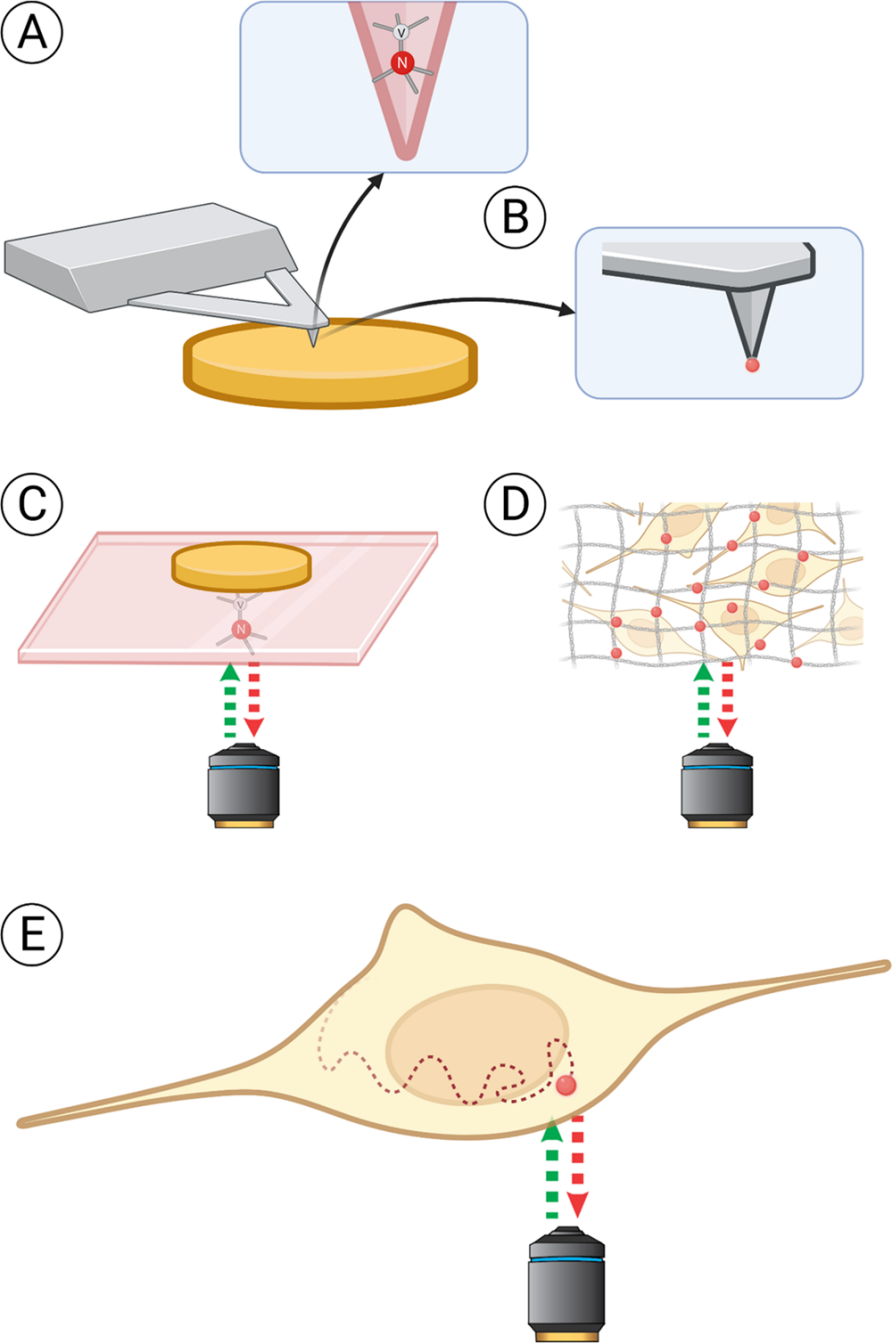
Different ways to conduct relaxometry experiments. The sample is shown in yellow, the (nano)diamond hosting NV centers in red. Three main approaches can be defined. In the first case (A, B), the sample is scanned with a NV-containing probe, similarly to atomic force microscopy. The scanning tip can either be made of diamond hosting NV centers (A) or have a nanodiamond attached to it (B). The second approach (C, D) relies on obtaining information from a stationary diamond placed close to the sample. Most commonly, bulk diamond plates are used for that (C). Nanodiamonds immobilized on a supporting surface or embedded in polymer matrices offer a cheaper alternative. They can be measured through a cover glass, in a microfluidic chip, or diluted in solution in high concentrations. Also, they offer shorter measurement times than bulk diamond plates due to shorter T1 time. However, with nanodiamonds there is less control over the orientation of the NV centers and the defect properties are usually worse (D). In the third approach (E), individual nanodiamonds diffusing in the sample (chemical solution or a live cell) are tracked during the experiment, allowing one to map the spatial differences or to choose a specific position for the measurements. Nevertheless, direct measurement of single nanodiamonds in solution without immobilization is challenging. In cells, FNDs are transported/diffuse relatively slowly or can be anchored to specific locations (e.g., mitochondrial membrane, cell membrane).^1,3^

Depending on the application there are different ways to conduct relaxometry experiments (Figure 3). Three ways can be differentiated: relaxometry in moving or stationary nanodiamonds, scanning magnetometry, and relaxometry in a stationary bulk diamond with a stationary sample on top. Table 1 summarizes the different options as well as their advantages and limitations.

**Table 1:** Summary of Different Ways to Conduct Relaxometry Experiments

method	diamond material	pros and cons	refs
scanning magnetometry	microfabricated diamond probe (Figure 3A)	+ highest spatial resolution	antiferromagnetic textures^14^
		+ high degree of control over the number and position of NV centers	spin waves^15^
		– requirement for elaborate diamond engineering	
	nanodiamonds attached to scanning probe (Figure 3B)	+ no elaborate diamond engineering	thermal and magnetic imaging^16^
		– poor control over NV position	
relaxometry on a stationary diamond	bulk diamond (Figure 3C)	+ best possible coherence time and thus sensing performance	temperature & magnetic field^11^
			picowells^17^
			chiton teeth^18^
		+ diamond surface can be structured to increase the surface area or create picoliter wells for small sample volumes	microfluidic device; thin cell section^19^
		– limited sample options	ferritin adsorbed on the diamond surface^20^
			electrical conductivity^21^
			thermally induced magnetic noise^22^
			magnetic NPs^23^
			more magnetic NPs^24^
			antiferromagnetic materials^25,26^
			spin waves^27^
			spin chemical potential^28^
	nanodiamonds (Figure 3D)	+ cheapest and simplest to get	ferritin adsorbed to ND surface^29^
			FNDs mixed with blood sample and dried on the glass^30^
		+ allows intracellular measurements	NDs in microfluidic device^31^
			NDs with responsive coating^32^
			ions in solution^33^
		– poor coherence times and defect properties	magnetic NPs^34^
			NDs embedded in extracellular scaffold^35^
			pH^36^
			pH, redox potential in a microfluidic channel^37^
			viral RNA–theoretical^31^
	diffusing nanodiamonds (Figure 3E)	+ freedom to choose the position within the sample/mapping the sample	radical generation^38^
			radical generation in sperm cells^3^
			free radicals in mitochondria^1^
		– changing location, need for tracking	free radicals during viral infection^4^
			free radicals in primary dendritic cells^2^
			polymer degradation^39^

## Relaxometry Applications

### Relaxometry in Cells

**Figure 4:**
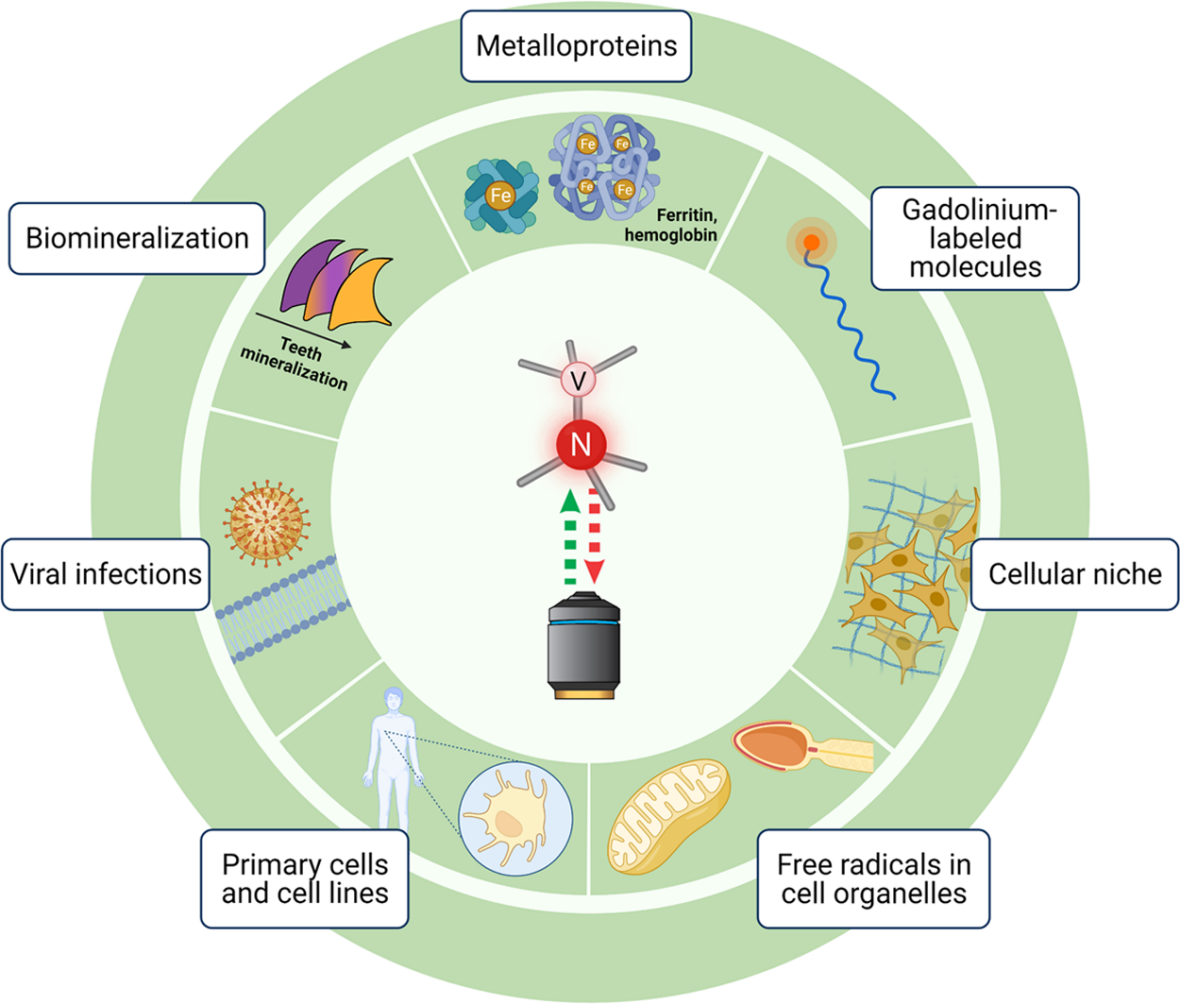
Applications of relaxometry for biomedical samples. Naturally occurring magnetic crystals, as well as metal ions incorporated in metalloproteins, are a source of magnetic signal that can be detected with NV-based sensing, often with bulk diamond plates. Paramagnetic species, such as gadolinium, can be used to label molecules and structures of interest for relaxometry measurements. Nanodiamonds hosting NV centers can be introduced in the cellular niche or even inside the cell for highly localized detection of magnetic signals, such as those produced by free radicals.

Recently relaxometry found a number of applications in biological and biomedical research (Figure 4). For an excellent, broader (not specific for relaxometry) overview of the main challenges and prospects of using diamonds with NV-centers for biologically relevant measurements, we refer the reader to two recently published reviews.^7,8^

Live cells are extremely complex systems, in which thousands of physiological processes take place simultaneously on various time scales, from microseconds to days.^40^ These processes both depend on and affect a wide range of physical and chemical parameters, such as pH, temperature, local concentrations of metabolites, and so on. The task of capturing and unraveling these networks becomes even more challenging, if one considers the interaction of a cell with its environment, including the neighboring cells. Moreover, the measurements have to be performed in a wet, warm (room temperature and higher), oxygen-rich environment with relatively high concentrations of salts, varying pH values, and the chance for the cell or the subcellular component of interest to actively move during the measurement. However, high-resolution quantitative detection of the molecular events at the single-cell and subcellular level is vital to understand cell behavior under normal or pathological conditions.

In biological samples, the magnetic signal that can be detected by relaxometry comes from different sources. Naturally occurring magnetites are found in a wide range of organisms, from magnetotactic bacteria^41^ and molluscs^42^ to humans.^43^ They are thought to be involved in the sensing of magnetic fields,^44^ as well as the development of some diseases,^45^ such as Alzheimer’s. Certain paramagnetic species, such as iron, copper, and manganese, as well as free radicals, are naturally ubiquitous in biological samples, whereas gadolinium is widely used for labeling. NV-center-based relaxometry has been used to detect the signals coming from each of these sources. Free radicals, gadolinium, and iron have already been detected in cells.

As mentioned earlier, relaxometry measurements can be performed with different forms of diamonds (Table 1). Another commonly used technique is to label the sample with gadolinium, an essential component of MRI contrast agents. It strongly affects the relaxation rate of NV centers, allowing one to visualize labeled cellular components.^46,47^

Very recently, NV-based relaxometry has found an application for *in situ* detection of free radicals. Free radicals contain at least one unpaired electron, which makes them paramagnetic. They play important roles in a vast range of physiological processes, including infections and immune response,^48^ cancer development and treatment,^49^ cellular signaling and communication,^50^ interaction of germ cells,^51^ and aging.^52^ First proof-of-concept studies focused on detecting and measuring free radical concentrations in a more controlled chemical setting.^32,33^ Embedding nanodiamonds in the components of the cellular niche or extracellular matrix offers a way to perform relaxometry in a more complex three-dimensional system.^35^ Moreover, excellent biocompatibility of nanodiamonds^53^ makes it possible to bring those tiny sensors inside a living cell. Cells carrying nanodiamonds can then be exposed to varying conditions, such as changing flow rates of the medium^38^ or certain drugs that affect cell function.^1,54^ Recently, also the free radical generation upon bacterial exposure to antibiotics has been measured.^55^ In this work, Norouzi et al. showed that the radical load on single bacteria increases with an increase in the dose of antibiotics. High spatial resolution allows detection of free radical production in a very localized way, at the level of a single bacterial cell or even specific organelles, such as mitochondria^1^ or acrosomes of sperm cells.^3^ High temporal resolution of the technique, on the other hand, provides insight into dynamic biological processes, such as free radical generation during viral infection.^4^ It is worth mentioning that it is possible to perform relaxometry measurements in stationary and to some extend even in mobile cells (as for instance moving sperm cells or migrating cancer cells). Furthermore, this technique requires relatively less biological material than other standard methods used for detection of paramagnetic species. This makes relaxometry particularly suitable for clinical settings,^2^ where collecting additional material for the measurements (biological fluids, tissue biopsies, or primary cells^2^) from the patient can be a challenging task. Nevertheless, when performing relaxometry measurements in living cells one should include controls that will prove that magnetic signal comes from a particular source. So far it has been done by inhibition or/and stimulation of well-known processes in which free radicals were generated by particular organelles.^1,3^ In order to further increase reliability of the method in biological samples, one should consider targeting and monitoring of the location of a FND as its sensing capability decreases with the distance from the source. Therefore, researchers have been using various functionalization strategies for nanodiamonds, especially with antibodies, in order to ensure delivery and then detection of magnetic signals from specific locations in cells.^1^ Moreover, researchers have shown that modification of surface chemistry of FNDs allows control over protein corona formation and therefore could worsen their sensing performance.

### Relaxometry for Applications in Material Science

Also in material science, relaxometry can offer some new insights. Probably the most prominent application in material science is in diamond engineering itself. In this context, relaxometry is used as a relatively simple tool to assess the quantum properties of new diamond materials. Generally, the longer the T1 of the material the more promising the diamond material is for quantum sensing applications. This approach has for instance been used by Tetienne et al. to evaluate^12^ the effects of the nanodiamond size on the inherent T1 relaxation time. Smaller diamonds show shorter T1 values, as the contribution of the surface paramagnetic centers becomes more prominent than in the bulk diamond.

Another application is to measure chemicals in solution. This way, it is possible to follow chemical reactions or measure the concentrations of certain paramagnetic species in solution.^56^ These can be for instance spin labels, MRI contrast agents, or other paramagnetic species like free radicals.^33^

Yet another application has been demonstrated by Li et al.^39^ The authors have used relaxometry to follow polymer degradation. To achieve this goal, they have incorporated nanodiamond particles for sensing into a biodegradable polymer. The polymer was then degraded in a medium that was spiked with gadolinium ions. When the polymer is intact, the Gd^3+^ is relatively far from the diamond. Once the polymer degrades, the Gd^3+^ reaches the particles and the relaxation time decreases.

Other applications that have been published include the characterization of conductive or magnetic materials. Electrons moving in a conductor give rise to magnetic fields, which affect the T1 relaxation rate of NV centers, allowing one to assess the local changes in conductivity of the target material at nanoscale resolution.^21,22^ As an example, Ariyaratne et al.^21^ monitored changes in conductivity of a metal structure with a scanning magnetometer. High spatial resolution is also attractive for materials that have a nanoscale magnetic structure or that are nanoscale objects. This approach has been used to detect and characterize magnetic nanoparticles,^16,18,24,34^ which exhibit superparamagnetic behavior at room temperature. Their superparamagnetic state can be detected by T1 relaxometry.

Another area that has benefited from diamond-based relaxometry is imaging of magnetic domains in antiferromagnetic materials, which are difficult to detect with conventional magnetometry. Following the theoretical proposal of Flebus et al.,^57^ several experimental studies on this topic have been recently published,^14,25,26^ using a scanning probe,^14^ a microscopic diamond prism placed on the surface of the material,^26^ or a diamond microchip allowing for wide-field relaxometry measurements.^26^

### Relaxometry in Physics

While in physics more complex pulsing sequences are more popular, there are a few applications where relaxometry has been utilized. High sensitivity of NV-based relaxometry makes it a powerful tool for measuring complex and dynamic physical processes at nanoscale resolution. Relaxometry has found exciting applications in condensed matter physics. A theoretical framework for using impurities in the diamond structure, such as NV or SiV centers, has been developed by Flebus et al.,^57,58^ and Van der Sar et al.^27^ have used NV centers in bulk diamonds to probe the spin noise in a ferromagnetic microdisc fabricated on the diamond surface. In another study, Du et al. measured spin chemical potentials, the tendency of spins to diffuse, in the magnetic insulator yttrium–iron–garnet film.^28^ Similarly, Simon et al. employed the diamond cantilever scanning system to characterize the magnon behavior in the same material with high local precision.^15^

### Detecting Other Quantities than Magnetic Noise

When performing relaxometry experiments, one should be aware that changes in T1 can be caused by various physical and chemical properties of the environment.^11^ While this can make the interpretation of the results more complicated, it also offers the possibility of multifunctional sensing. One of the earlier relaxometry studies employed a nanodiamond attached to the AFM tip to perform nanoscale magnetic and thermal (in the range of 290–330 K) imaging.^16^

Jarmola et al.^11^ have studied the temperature and magnetic dependences of the T1 in NV^–^ ensembles of bulk diamonds. They have shown that two-phonon Raman- and Orbach-type processes are the main relaxation mechanisms. This study also revealed temperature independent longitudinal relaxation at low temperatures. Under such conditions the T1 relaxation is magnetic field dependent and strongly affected by the cross-relaxation between differently aligned NV-centers as well as between NV-centers and substitutional nitrogen impurities known as the P1 centers. Similarly, de Guillebon et al. have investigated the relaxation mechanism of single NV centers in nanodiamonds at both room and cryogenic temperatures.^59^ Their results have shown that the temperature-dependent relaxation process is attributed to a thermally activated magnetic noise produced by paramagnetic impurities lying on the nanodiamond surface.

It is known that T1 of carboxylated diamonds is slightly affected by the changes in pH in the range of 3–7, with lower pH resulting in longer T1.^36^ Viscosity had a relatively negligible effect on T1 measurements.^1^ Additional coating of the diamond surface with functional groups, such as poly(l-cysteine), can shift the pH sensitivity to the range between 7 and 11.^36^ Diamonds with an untreated surface, which has a graphite layer and a number of various functional groups, do not show pH dependence of T1.

**Figure 5:**
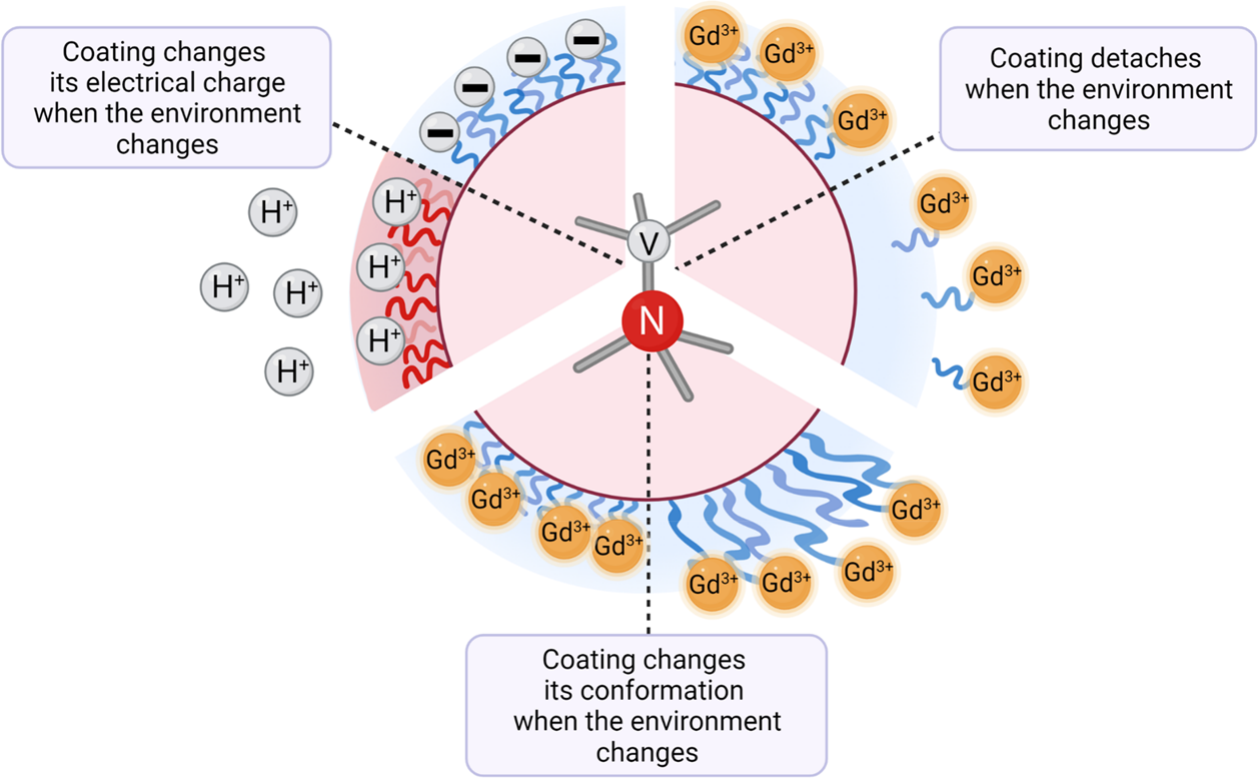
Coatings that respond to the changes in the environment can broaden the range of applications for relaxometry measurements. Coatings that change their charge in a specific pH range (e.g., poly(l-cysteine) with p*K*_a_ = 8–10) will make the NV centers in the diamond sensitive to that range of pH changes. Gadolinium-labeled coatings that detach or expand upon stimulus bring the paramagnetic label closer to or further away from the diamond surface, directly affecting T1 values.

An attractive way to extend the usefulness of relaxometry measurements is to synthesize responsive coatings (Figure 5). The principle behind these coatings is that they contain detectable species (for instance, gadolinium or spin labels) that are attached to the surface via a responsive molecule, which is, for instance, cleaved under certain conditions. This results in the label diffusing away from the particle, which in turn causes an increase in T1. It is also possible to use responsive molecules, which shrink or extend upon a stimulus. This way the label is closer (low T1) or further away (high T1) from the diamond surface. This approach has been used to make nanodiamond-based sensors for pH (in the range of 2–7.4) or redox potential, depending on the responsive linker that was used for coating.^37^ This technique was recently extended for highly sensitive detection of biomarkers, such as viral RNA. In this case, the diamond surface is coated with complementary DNA, which is, in turn, labeled with gadolinium. If target RNA is present in the environment, the DNA molecules will bind it and detach from the diamond surface, carrying away the gadolinium label and causing a shift in T1.^31^ The drawback of cleavable coatings is that the responsive molecule is irreversibly removed from the surface, so the sensor cannot be reused or used to detect the opposite change of the environment at a later time point. Platforms based on the linkers that change their conformation without detaching from the diamond surface could solve this problem.

## Conclusion

Relaxometry offers a highly sensitive readout for magnetic noise. As a result of this unprecedented sensitivity, the technique allows nanoscale spatial resolution as well as temporal resolution in the minute range. Additionally, relaxometry only requires low sample volumes and is thus compatible with single cell or even single organelle measurements. A further advantage of relaxometry measurements is that they allow reversible read out in real time.

Due to the simplicity and low cost of the approach (only optical access is needed and the technique works at room temperature) relaxometry is very versatile and has been implemented in many different instruments and for a wide range of applications.

However, there are still challenges that have to be resolved in order to make use of the full potential of relaxometry. Influence by other parameters, most notably pH, on T1 is problematic. Additionally, it is imperative that there is optical access, and the (nano)diamonds have to be within a few nanometers from the sample. Finally, there is a great variability between NV centers. In some cases this is not a problem because a good NV center can be reused indefinitely (until the probe is broken or the NV is lost). However, especially in biological applications this is often not an option. In this case, large ensembles can be used to reduce variability. However, the field would still gain from diamonds with more uniform defects as well as size and shape.

## Abbreviations

NV center: nitrogen vacancy center
MRI: magnetic resonance imaging
FND: fluorescent nanodiamond
RNA: ribonucleic acid
SiV: silicon vacancy
AFM: atomic force microscopy
CCR2: CC chemokine receptor 2
CCL2: CC chemokine ligand 2
CCR5: CC chemokine receptor 5
TLC: thin layer chromatography
